# Multistate Outbreak of Human *Salmonella* Infections Linked to Live Poultry from a Mail-Order Hatchery in Ohio — February–October 2014

**Published:** 2015-03-13

**Authors:** Colin Basler, Tony M. Forshey, Kimberly Machesky, C. Matthew Erdman, Thomas M. Gomez, Denise L. Brinson, Thai-An Nguyen, Casey Barton Behravesh, Stacey Bosch

**Affiliations:** 1 Epidemic Intelligence Service, CDC; 2 Division of Foodborne, Waterborne, and Environmental Diseases, National Center for Emerging and Zoonotic Infectious Diseases, CDC; 3 Ohio Department of Agriculture; 4 Ohio Department of Health; 5 US Department of Agriculture

In early 2014, five clusters of human *Salmonella* infections were identified through PulseNet, the national molecular subtyping network for foodborne disease surveillance. Many ill persons in each of these clusters reported contact with live poultry, primarily chicks and ducklings, from a single mail-order hatchery; therefore, the clusters were merged into a single investigation. During February 3–October 14, 2014, a total of 363 persons infected with outbreak strains of *Salmonella* serotypes Infantis, Newport, and Hadar were reported from 43 states and Puerto Rico, making it the largest live poultry-associated salmonellosis outbreak reported in the United States.

Among the ill persons, 35% (122 of 353) were aged ≤10 years, and 33% (76 of 233) were hospitalized; no deaths were reported. Among those interviewed, 76% (174 of 230) reported live poultry contact in the week before illness onset. Among the ill persons who provided supplemental information on live poultry exposure, 80% (94 of 118) reported chick exposure and 26% (31 of 118) reported duckling exposure. Among 96 (81%) ill persons who were exposed to live poultry at their residence, 28 (29%) reported keeping poultry inside their home instead of outdoors, and 26 (27%) reported no direct contact with their poultry.

Of the 75 ill persons with live poultry purchase information, the average time from purchase to illness onset was 48 days (range = 2–730 days); 27 (36%) reported illness onset within 14 days of purchase. Hatchery source information was available for 69 purchases, of which 58 (84%) came from a single mail-order hatchery in Ohio. This same Ohio hatchery was previously linked with multiple, large human *Salmonella* infection outbreaks (1,2).

The U.S. Department of Agriculture’s National Poultry Improvement Plan, a collaboration between industry and state and federal agencies, provides guidance on management and sanitation practices for mail-order hatcheries, including a Best Management Practices Handbook.* Comprehensive *Salmonella* prevention and control programs are needed at all hatcheries and associated breeder farms to prevent outbreaks.

The possibility of environmental contamination of the home by live poultry, suggested by the 27% of respondents who reported no direct contact with their poultry, illustrates a need for additional educational information advising customers on how to reduce the risk for *Salmonella* transmission from live poultry (3) to humans through environmental contamination. Educational information regarding zoonotic *Salmonella* outbreaks, including outbreaks associated with live poultry, is available from CDC (4). A comprehensive approach to illness prevention involving human and animal health officials and practitioners, industry, and backyard poultry flock owners is needed to prevent future outbreaks.

## Footnotes

* Available at http://www.poultryimprovement.org/default.cfm.
